# High Throughput Preparation of Poly(Lactic-Co-Glycolic Acid) Nanoparticles Using Fiber Fluidic Reactor

**DOI:** 10.3390/ma13143075

**Published:** 2020-07-09

**Authors:** Niloofar Heshmati Aghda, Emilio J. Lara, Pulinkumar Patel, Tania Betancourt

**Affiliations:** 1 Materials Science, Engineering and Commercialization Program, Texas State University, San Marcos, TX 78666, USA; n_h137@txstate.edu; 2 Department of Chemistry and Biochemistry, Texas State University, San Marcos, TX 78666, USA; ejl38@txstate.edu (E.J.L.); pulinpatel71@outlook.com (P.P.)

**Keywords:** nanoprecipitation, nanoparticles, polymers, poly(lactic-*co*-glycolic acid) (PLGA), process optimization, fiber reactor, large scale, high throughput preparation

## Abstract

Polymeric nanoparticles (NPs) have a variety of biomedical, biotechnology, agricultural and environmental applications. As such, a great need has risen for the fabrication of these NPs in large scales. In this study, we used a high throughput fiber reactor for the preparation of poly(lactic-*co*-glycolic acid) (PLGA) NPs via nanoprecipitation. The fiber reactor provided a high surface area for the controlled interaction of an organic phase containing the PLGA solution with an aqueous phase, containing poly(vinyl alcohol) (PVA) as a stabilizer. This interaction led to the self-assembly of the polymer into the form of NPs. We studied operational parameters to identify the factors that have the greatest influence on the properties of the resulting PLGA NPs. We found that the concentration of the PLGA solution is the factor that has the greatest effect on NP size, polydispersity index (PDI), and production rate. Increasing PLGA concentration increased NP sizes significantly, while at the same time decreasing the PDI value. The second factor that was found to affect NP properties was the concentration of PVA solution, which resulted in increased NP sizes and decreased production rates. Flowrates of the feed streams also affected NP size to a lesser extent, while changing the operational temperature did not change the product’s features. In general, the results demonstrate that fiber reactors are a suitable method for the large-scale, continuous preparation of polymeric NPs suitable for biomedical applications.

## 1. Introduction

Polymeric nanoparticles (NPs) in the size range of 10–200 nm are suitable for biomedical applications that require local or systemic administration, interaction with diseased tissues at the cellular and molecular level, and uptake into cells. The high surface area and chemical versatility of these NPs enable surface functionalization, with targeting ligands that can enhance transport across physiological barriers and provide specificity toward molecular targets characteristic of diseased tissues. At the same time, because of their macromolecular size, NPs can readily act as carriers for controlled delivery of therapeutic agents, contrast agents or other cargo. As such, NPs are expected to be critical for future diagnostic, therapeutic and theranostic technologies [1].

In the field of nanomedicine, polymeric NPs have mostly been used for drug delivery approaches to facilitate the pulmonary, oral, transdermal and intravenous delivery of therapeutic agent for the treatment of cancer, infection diseases, and inflammation diseases [2,3,4,5]. While to date, only a few NP-based systems have entered the market as therapeutics or biotechnological tools, it is expected that nanotechnology will revolutionize health care in the near future [6]. Several NP-based formulations have been approved by the FDA, including liposomal doxorubicin (Doxil), liposomal daunorubicin (DaunoXome), liposomal amphotericin B (Abelcet, Amphotec, and AmBisome), and paclitaxel-loaded albumin NPs (Abraxane). Despite the high versatility and long history of polymeric materials in medicine, to date, no therapeutic nano-scaled NP formulations based on biodegradable polyesters such as poly(lactic-*co*-glycolic acid) (PLGA) have been approved by the FDA for clinical use.

As diagnostic agents, polymeric NPs can be used as contrast agents for biomedical imaging, labeling probes for biomarker or pathogen detection, or as capture agents for the separation of biological molecules or cells [7]. Conjugates of polymeric NPs with antibodies, aptamers and oligonucleotides enable the detection of the disease biomarkers [8]. NPs can also be incorporated into biomedical device coatings or blended as nanocomposites for the preparation of drug eluting stents, tissue engineered scaffolds, or antibacterial coatings that require the controlled release of active agents, high porosity, or nano-scaled topologies.

Polymeric NPs have also been used for separation and purification in bioprocesses [8]. Stimuli-responsive NPs have also been used as nanofillers to provide tunable porosity within gels for the separation of biological molecules through electrophoresis [9]. Polymeric NPs have also been utilized in the cosmetic industry for the delivery of skin care, antiacne and antioxidant agents to the pores of the skin [10]. Highly permeable hair products based on polymer NPs are being fabricated to deliver blood flow acceleration, cell activation and androgen suppression agents [10]. The food industry can also benefit from polymer-based NPs for the encapsulation of phytonutrients and prebiotics [11]. The use of polymeric NP has been reported in environmental applications, for instance, in the bioremediation of soil [12].

Several methods have been developed to prepare polymeric NPs, including nanoprecipitation, emulsion-diffusion methods, emulsion-evaporation, precipitation by salting-out, and polymerization [13]. Amongst all, nanoprecipitation, also known as solvent displacement method, has gained the most attention, since it is a simple, fast, inexpensive, one-step process. Nanoprecipitation occurs due to the interfacial deposition of the polymer led by the introducing a non-solvent, which is miscible with the solvent [14]. Several studies have reported the scale-up of this process using different types of reactors, including impinging jets, single turbulent jets, rotor-stator mixers, static mixers stirred tank centrifugal pumps, and turbulent pipes [15,16,17,18]. Although favorable results have been reported through these methods, providing homogeneous yet fast mixing, which directly affects product characteristics such as size, and polydispersity index (PDI) has remained an important limitation. Similarly, recently developed microfluidic processes enable very reproducible preparation of precursor droplets, and consequently NPs [19]. However, the throughput of microfluidic methods prevents their direct adaptation into large-scale manufacture. Thus, the development of new technologies, that could enable the preparation of polymeric NPs in large scales through continuous processes, is needed.

Fiber reactors, or Fiber Film^®^ Contactors (Merichem Company, Houston, TX, USA), were first introduced in the 1970s, as mass transfer technologies for multi-phase processes in the petrochemical industry [20]. In this work, we utilized a high-throughput fiber reactor that we previously utilized for the preparation of NPs through a polymerization method [21], to produce NPs via nanoprecipitation using the model polymer poly(lactic-*co*-glycolic acid) (PLGA), which is commercially available and inexpensive, and has been widely used in drug delivery and bioimaging applications [22,23,24,25]. PLGA NPs have also been demonstrated to exhibit hemocompatibility, making them favorable candidates for biomedical applications [26]. The reactor is made of a stainless-steel tube packed with a plurality of unidirectional stainless-steel fibers, which provide a large surface area that the nonsolvent (an aqueous phase) can be spread over in a very thin film, and interface the polymer solution when it is introduced to perform nanoprecipitation. The main aim of the work is to elucidate the reactor parameters that control NP characteristics to enable the on-demand production of NPs with desired properties, in a continuous process with high efficiency. The possibility of loading active agent mimics through this method was further investigated by loading a model dye, rhodamine 6G, within the formed PLGA NPs.

## 2. Materials and Methods

### 2.1. Materials

PLGA (Purasorb PDLG 5002A, 50/50 DL-Lactide/Glycolide copolymer) was a gift from Corbion (Amsterdam, Netherlands). Poly(vinyl alcohol) (PVA) (87–90% hydrolyzed, avg. mol wt. 30,000–70,000 Da) was obtained from Sigma Aldrich (St. Louis, MO, USA). Rhodamine 6G was purchased from Coherent (Santa Clara, CA, USA). ACS grade acetone was used. A Millipore Direct Q system (Millipore, Burlington, MA, USA) produced ultrapure deionized water.

### 2.2. Reactor Set up

**Figure 1:**
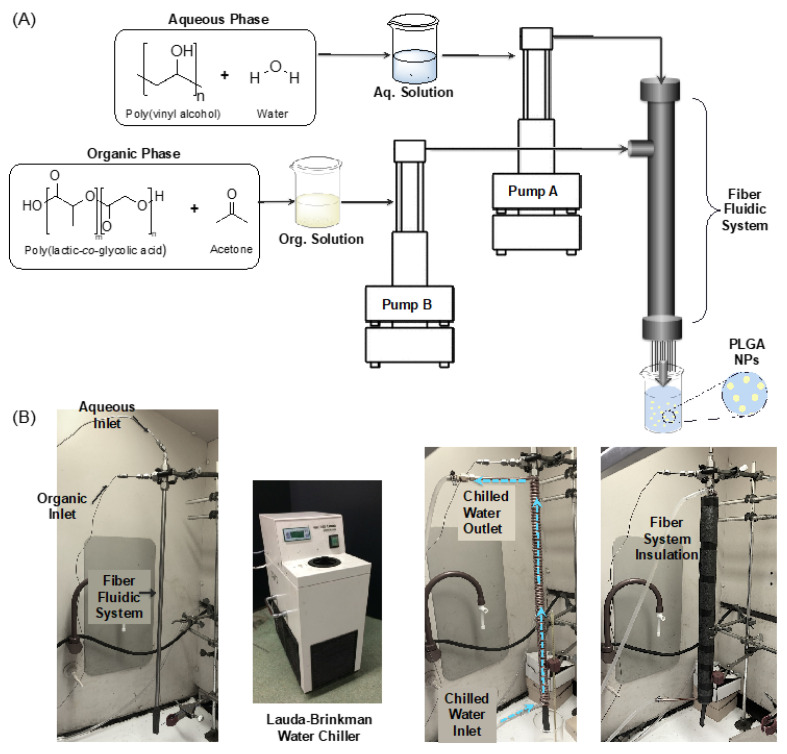
Fiber reactor setup. (**A**) Schematic of experimental fiber fluidic system setup. The fiber fluidic system has two inlets that are fed with syringe pumps. The aqueous phase, including poly (vinyl alcohol) (PVA) as a stabilizer, enters the system first, wetting the fibers. The organic phase, containing the polymer poly(lactic-*co*-glycolic acid) (PLGA) and active agents, is fed downstream. Nanoparticle (NP) suspensions exit the fiber reactor through the outlet, which is open to atmospheric pressure. (**B**) Left to right: Images of the bare fiber fluidic system, water chiller, fiber fluidic system wrapped with cooling coil, and fiber fluidic system with cooling coil and insulation.

A 2-ft long, cylindrical stainless steel reactor of 9.09-mm inner diameter and 11.65-mm outer diameter packed with 8-µm diameter stainless steel fibers at a packing density of about 8325 fibers/mm^2^ was used in this study. Two syringe pumps (Teledyne ISCO, Lincoln, NE, USA, Model 260D) with capacities of 100 mL and 270 mL were connected to the reactor input ports to feed the reactor. The pumps can either work simultaneously or independently. The reactor was surrounded by a copper cooling coil containing circulating water at set temperatures, which is provided by a WKL 230 LAUDA Brinkmann chiller (LAUDA-Brinkman, Delran, NJ, USA). The cooling coil was insulated with a polymeric foam to improve cooling efficiency. The product was collected from the reactor’s output at the bottom. Figure 1 shows the described setup.

It should be noted that, while a reactor with a 9.09-mm inner diameter was used in this study, industrial production from fiber reactors or Fiber Film^®^ contactors (Merichem Company, Houston, TX, USA) is expected to increase linearly with the cross-sectional flow area (i.e., with the reactor diameter squared), as long as fiber packing and feed velocities are maintained constant, in accordance with increasing reactor diameter. In addition, it is also important to know that there are additional parameters related to the fiber reactor design (diameter, length, packing density, fiber surface chemistry, etc.) that could be utilized to further effect NP production. In this present study, however, we focused on investigating the effect of process parameters on NP production, while maintaining the fiber reactor set up constant.

### 2.3. Fabrication of PLGA NPs Using the Fiber Reactor

PLGA NPs were made through nanoprecipitation. Briefly, syringe pumps A and B were filled with a 5–25 mg/mL aqueous solution of PVA, which is used as a stabilizer, and a 2.5–15 mg/mL solution of PLGA in acetone, respectively. Pumps were turned on at the same time to start feeding the reactor simultaneously at set flowrates while the chiller was operating at constant temperature (3 °C–15 °C). The output stream, which contained the formed NPs, was collected and used for further studies. The temperature of the output flow was also recorded by a TM902C digital LCD K Type thermometer (Lutron Electronic, Coopersburg, PA, USA). After each production process, pumps A and B were refilled with pure acetone and pure water, to clean the reactor by purging water and acetone, respectively. Collected NPs were centrifuged for 1 h at 75,600× *g* (Avanti J-26 XPI, Beckman Coulter, Brea, CA, USA), to remove acetone and excess surfactant.

### 2.4. Loading Rhodamine 6G within PLGA NPs Using the Fiber Reactor

To confirm the possibility of drug loading using the fiber reactor, we prepared rhodamine 6G-loaded PLGA NPs in the fiber reactor. Pump B fed a 5 mg/mL PLGA solution in acetone containing 0.01 mg/mL rhodamine 6G to the reactor at 3.5 mL/min, while pump A fed a 10 mg/mL PVA aqueous solution at 25 mL/min. The cooling coil was maintained at 10 °C.

### 2.5. NP Size and PDI Measurement

NP size and polydispersity index (PDI) were determined by dynamic light scattering using a Malvern ZetaSizer Nano ZS instrument (Malvern Panalytical, Westborough, MA, USA). Measurements were done at 24 °C, and the PVA solution was selected as the dispersant. The viscosity and refractive index (RI) of PVA solutions at specific concentrations matching each sample were measured before sizing, using a glass viscometer and refractometer (data not shown). These values were input to the ZetaSizer software for proper data analysis.

### 2.6. Morphology of NPs

To investigate their morphology, the NPs were deposited on silicon wafers, dried at room temperature, and coated with 2 nm of iridium using a Quorum Technologies EMS150T ES sputter coater. NPs were then visualized using a FEI Helios NanoLab 400 Dual Beam scanning electron microscope (SEM, FEI, Hillsboro, OR, USA).

### 2.7. Yield Measurement

First, 40 mL aliquots of NPs were centrifuged for 1 h at 75,600× *g* (Avanti J-26 XPI, Beckman Coulter, Indianapolis, IN, USA). Then, the supernatant was discarded, and NPs were resuspended in water and lyophilized (Labconco FreeZone, Labconco Corporation, Kansas City, MO, USA) in pre-weighed vials. The net weight of NPs was measured after lyophilization, and the rate and yield of production were calculated.

### 2.8. Statistical Analysis

Three independent experiments (batches) were conducted to produce NPs for each set of process conditions. For each of these batches, four samples of the output from the reactor were obtained at different time points, starting at 5 min of continuous flow. The size and PDI of the NPs produced in each of the three replicate experiments (12 data points per condition) were averaged, and their standard deviations were calculated. Similarly, the production rate and conversion yield of each of the three replicate experiments (3 data points per set of conditions) were averaged, and their standard deviations were calculated. The plots show the averages for each condition and with standard deviations as error bars. Student t-tests were utilized to identify statistical differences between conditions.

## 3. Results and Discussion

Nanoprecipitation occurs due to the nucleation of the polymer and growth of the nuclei, which can either end up with the aggregation of the NPs or the stabilization of the NPs mediated by a stabilizer such as a surfactant [27]. The nucleation and growth rates, which directly influence NPs size, are controlled by saturation. Saturation is defined as:

(1) $$S=\frac{C}{C^{*}}$$

where *C* is the real-time concentration of the solute, and C* is the solubility of the solute in the mixed solution. When the mixing happens gradually, *C* and C* gradually change; thus, the level of saturation varies over time until full mixing is achieved. This means that the NPs that are formed in this period are affected by changing conditions, resulting in a variety of sizes and, thus, broad size distribution. On the other hand, if the mixing time is reduced to an amount less than the time which is required for NP formation, all the NPs form under the same saturation conditions, leading to uniform NPs with low PDI [28]. Therefore, mixing time in nanoprecipitation has a crucial role, and it is an important issue to consider when the process is scaled up. In large-scale nanoprecipitation, the focus is on keeping the Damköhler number less than 1 by rapid mixing of the nonsolvent with the polymer solution. The Damköhler number for nanoprecipitation is defined as:

(2) $$Da_{p}=\frac{\tau_{mix}}{\tau_{f}}$$

where *τ_mix_* and *τ_f_* are the mixing time and formation time of NPs, respectively.

**Figure 2:**
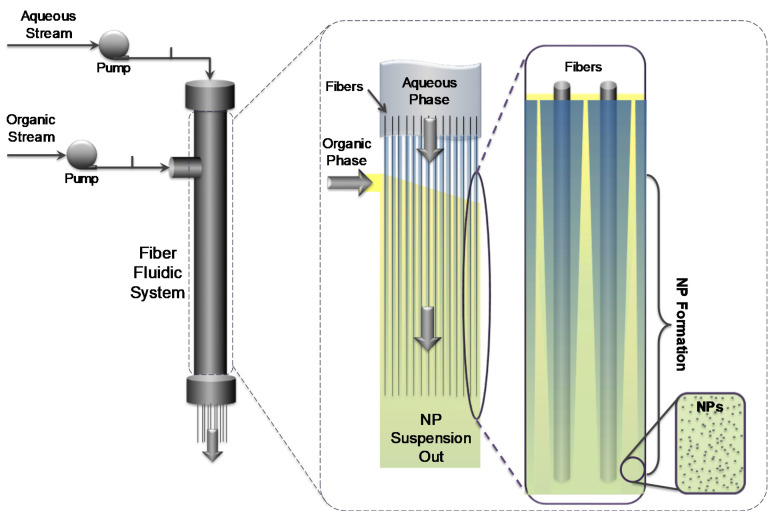
Schematic of an inside cross section of the reactor.

In our system, when the feed streams are introduced to the fiber reactor, they are divided into a plurality of sub-streams with a diameter of a few µm [21,29]. Figure 2 shows a scheme representing the phase distribution inside the reactor. The flows occupy and share the void area between the fibers, based on the ratio of their flow rates. The aqueous flow is expected to be in touch with the hydrophilic stainless-steel fibers, while the organic phase is in the middle, focused in between the aqueous streams. Thus, the maximum diffusion path is half of the distance between two fibers, which is W_d_ = 1.09 µm. Assuming unidirectional mass transfer (from the organic phase to the aqueous phase) and, according to Fick’s second law of diffusion, the required time for the complete mixing is calculated to be in the range of less than 1 ms, depending on the volume ratio of the organic to aqueous phases (see Figure S1 and derivation in the Supplementary Materials). As long as this value is less than the aggregation time of the polymer, the reactor’s products are expected to be uniform.

NP formation, as earlier discussed, includes the nucleation and growth of the polymer aggregates under saturation. Therefore, the rate of NP formation can be estimated according to the classical theory of nucleation [30]. The nucleation rate of the polymer per volume unit and time unit is defined as:

(3) $$J\;={\;N}_{0}{\;\upsilon \;e}^{-\frac{16{\pi \sigma }^{3}V_{s}^{2}}{3k^{3}T^{3}{\left(LnS\right)}^{2}}}$$

where *S* is the degree of saturation, N_0_ is the initial number of molecules of solute per unit volume, υ is the frequency of molecular transport to the solid-liquid interface, σ is the interfacial tension at the solid-liquid interface, vs. is the volume of a solute molecule, k is the Boltzmann constant, and T is the temperature. The frequency of molecular transport can be estimated by:

(4) $$\upsilon =\frac{\mathrm{kT}}{3\pi a^{3}\eta }$$

where η is the viscosity of the solution and “a” the mean effective diameter of the diffusing species [31]. In general, Equation (3) suggests that, as the degree of saturation decreases, the rate of nucleation and NP formation decreases. For a specific mixing time, *τ_mix_*, a decreased rate of nucleation would result in an increased NP formation time, *τ_f_*, and thereby a decreased *Da_p_*. Therefore, the NP properties are controlled by the formation process as opposed to the mixing quality.

### 3.1. Effect of PLGA Copolymer Concentration

**Figure 3:**
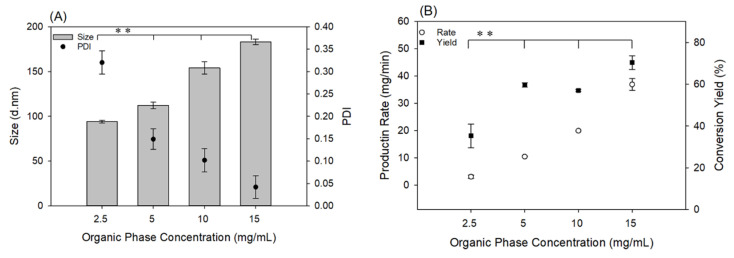
Effect of PLGA concentration on (**A**) NP size and PDI and (**B**) production rate and conversion yield. For this study, the flowrate of the PLGA solution (organic stream) was 3.5 mL/min, and the concentration and flowrate of the PVA solution (aqueous stream) was 10 mg/mL and 25 mL/min, respectively. ** Statistically significant difference in the size, PDI, production rate and conversion yield among all organic phase concentrations tested (*p* < 0.05).

Figure 3 shows the effect of the polymer concentration on NP size and PDI. PDI values decreased as the PLGA concentration increased. Due to the lower degree of saturation in lower concentrations, the NP formation time increases. It means that more time is required for the nuclei to grow and reach a thermodynamic equilibrium state. Therefore, agglomeration of the unequilibrated nuclei which formed in this time occurs. This is the reason why PDI increases when the feed polymer concentration decreases. However, increasing the concentration of the polymer solution led to the formation of larger NPs. This observation can again be explained by the effect of saturation on the nucleation rate per Equation (3), and to a greater importance, on the growth rate. Higher concentration leads to higher S, which causes a higher rate of nucleation and growth [32]. At a higher saturation level, a greater extent of the polymer tends to precipitate out of the solution phase, which provides the required NP building units for the nuclei to growth. Figure 3B shows the variation of production rate and yield percent versus PLGA concentration. Here, production rate refers to the recovery rate of formed and centrifuged NPs, in mg/min. As expected, adding more polymer in the feeding stream results in a higher production rate, which is the result of the higher nucleation rate (higher number of NPs), as well as the higher growth rate (more massive NPs).

As Figure 3B shows, the production rate ranged from 3.1 ± 0.6 mg/min (4.5 g/day) to 36.9 ± 2.1 mg/min (53 g/day) depending on the organic phase concentration with the current fiber reactor setup, while maintaining the average size of the NPs below 200 nm. As mentioned earlier, production can be readily scaled up with increased reactor diameter, thereby making this an industrially feasible continuous process. For example, the 53 g/day scale mentioned above could readily scale to 625 g/day, by utilizing a reactor of 5-cm nominal diameter instead of the 1-cm diameter utilized in this study. Similar production capacity has been reported for on-scale nanoprecipitation of amphiphilic block copolymers, including those with PLGA blocks, using novel automated coaxial jet mixer systems [33]. In comparison, laboratory scale batch processes typically result in the preparation of 1–100 mg of NPs per batch [22,34], and microfluidic systems produce NPs in the order of a few mg per hour [35].

### 3.2. Effect of Operation Temperature

**Figure 4:**
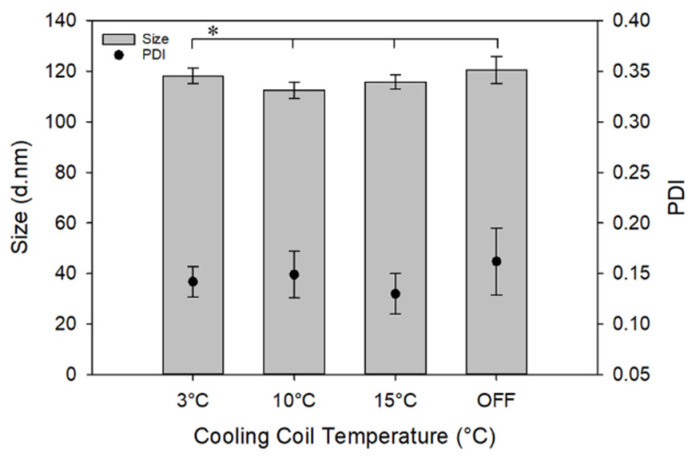
Effect of temperature of cooling coil on NP size and PDI. For this study, concentration and flowrate of the PLGA solution (organic stream) was 5 mg/mL and 3.5 mL/min, and that of the PVA solution (aqueous stream) was 10 mg/mL and 25 mL/min, respectively. * No statistically significant difference in the size or PDI of NPs as a function of cooling coil temperature (*p* > 0.05).

As previously explained, the reactor temperature was controlled by circulating water through a copper coil wrapped around the fiber reactor. The organic feed was kept at room temperature before running the reactor, while the aqueous feed was input at 4 °C. Figure 4 shows the effect of operating temperature on the NPs’ size and PDI. Neither the NP diameter nor the PDI changed upon changing the temperature of the cooling coil, although, based on Equation (3), a significant effect of temperature on size was expected. There are a couple of reasons that could explain the lack of diameter variation with temperature. For instance, temperature not only affects the nucleation rate, but also influences the saturation concentrations, diffusion rates, and the solution’s viscosity. Thus, these contrasting effects may have been neutralized and may have led to the experimental observation. This observation is noteworthy from an industrial standpoint, since we can eliminate a significant source of energy consumption and instrumentation for temperature adjustment.

### 3.3. Effect of The Ratio of the Flow Rates (Organic Stream/Aqueous Stream)

**Figure 5:**
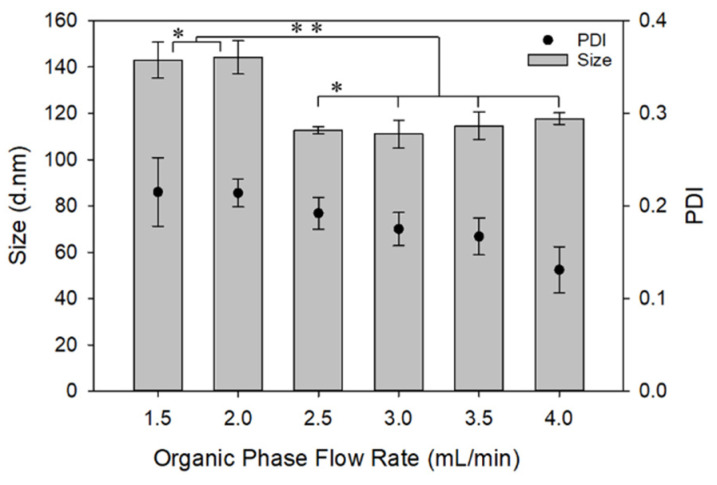
Effect of the flowrate ratio on NP size and PDI. For this study, the concentration and flowrate of the PVA solution (aqueous stream) was 10 mg/mL and 25 mL/min, respectively. The concentration of PLGA solution was 5 mg/mL, and its flowrate varied from 1.5 to 4 mL/min. * No statistically significant difference in the size of the NPs prepared with 1.5 or 2.0 mL/min organic phase flow rate, or between the NPs prepared with 2.5 to 4.0 mL/min organic phase flow rate (*p* > 0.05). ** Statistically significant difference between the size of NPs prepared with 1.5–2.0 mL/min and those prepared with 2.5–4.0 mL/min organic phase flow rate (*p* < 0.05).

As Figure 5 shows, by increasing the solvent/non-solvent flowrate ratio from 0.06 (1.5 mL/min organic vs. 25 mL/min aqueous flow rates) to 0.1 (2.5 mL/min organic vs. 25 mL/min aqueous flow rates), NPs’ size decreased, but stayed constant as the organic flow rate and, thereby, the organic/aqueous ratio further increased. A decrease in NP size was expected, as the ratio of solvent/non-solvent volume increased as a result of decreased saturation, and thereby decreased nucleation rate and NP growth per Equation (3). However, increasing the solvent/non-solvent flowrate ratio not only increases the amount of the polymer in the final reactor output, but also increases the total volume of the mixture. Thus, although the saturation degree decreases as the solvent/non-solvent flow rate ratio increases, the change is slight and the effect on NP size may thereby not as pronounced as expected. It is also possible that smaller NPs produced in batches with high solvent/non-solvent ratio are less thermodynamically stable and aggregate within the length of the fiber reactor, thereby somewhat neutralizing the effect of saturation on NP size once a certain solvent/non-solvent ratio is reached. This, together with accompanying changes in solution viscosity and diffusivity with the changing solvent/non-solvent ratios, could have led to the confounding effect observed.

### 3.4. Effect of PVA (Stabilizer) Concentration

**Figure 6:**
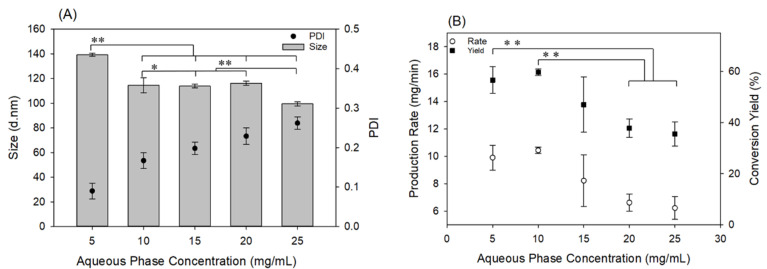
Effect of PVA concentration on aqueous phase on (**A**) NP size and PDI and (**B**) production rate and conversion yield. For this study, the flowrate of the PVA solution (aqueous stream) was 25 mL/min, and the concentration and flowrate of the PLGA solution (organic stream) was 5 mg/mL and 3.5 mL/min, respectively. In (**A**): * No statistically significant difference between the size of NPs prepared with the aqueous phase concentration at 10, 15, or 20 mg/mL (*p* > 0.05). ** Statistically significant difference in the size of NPs prepared with the aqueous phase at 5 mg/mL, and all higher aqueous phase concentrations, as well as between that of the NPs prepared with the aqueous phase at 25 mg/mL and at the lower aqueous phase concentrations (*p* < 0.05). In (**B**): ** Statistically significant difference in the production rate and conversion yield of NPs prepared with aqueous phase concentration at 5 and 10 mg/mL vs. that of NPs prepared with 20 and 25 mg/mL.

The stabilizer comes to part at the end of the nanoprecipitation process and stops further growth of the NPs. So, as the concentration of the stabilizer, which is PVA in this study, increases, NP size decreases. This fact is confirmed by our data presented in Figure 6A. The presence of a stabilizer is required to control NP size and prevent aggregation. However, as the data in Figure 6B show, the production rate decreases with increasing PVA concentration. While this might simply be a result of decreased recovery rate via centrifugation, for further industrial designs, this factor should be balanced to achieve both optimized production rate and size.

### 3.5. Loading of Mimic Payload

**Figure 7:**
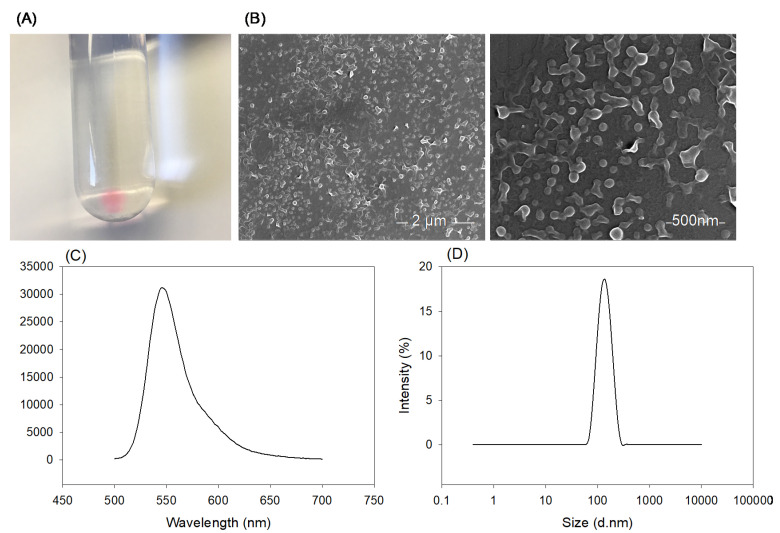
(**A**) Image of Rhodamine 6G-loaded PLGA NPs upon centrifugation, confirming their loading with the dye. (**B**) SEM images, (**C**,**D**) fluorescence spectrum (λ_EX_ = 480 nm), and size distribution of Rhodamine 6G-loaded PLGA NPs.

Rhodamine 6G was the model payload that we used to load within the PLGA NPs on a large scale using the fiber reactor. Figure 7A shows the centrifuged NP pellet, clearly showing entrapment of rhodamine with the polymer NPs from the pink color. The morphology of the resulting NPs is shown in Figure 7B, SEM images of the NPs. These images confirm the uniformity of produced NPs. Figure 7C shows the fluorescence spectrum of the NPs. The presence of the peak at 548 nm in the fluorescence spectrum confirms that the loading was successful. As Figure 7D shows, rhodamine loaded NPs had a narrow size distribution similar to that obtained with blank PLGA NPs. The average size and PDI of these NPs were 123.94 ± 2.02 nm and 0.130 ± 0.018, respectively. Incorporating rhodamine 6G into the NPS led to a slight size increase compared to the blank NPs that were prepared under the same conditions (i.e., equal concentration and flowrate of organic and aqueous streams), which had an average size of 112.48 ± 3.29 nm and a PDI of 0.149 ± 0.023. The slight change in PDI was not statistically significant (*p* > 0.05).

## 4. Conclusions

In this work, we introduced a facile and efficient process to make polymeric NPs in a continuous process through nanoprecipitation, which is one of the most common methods, utilizing a fiber reactor system. The fiber reactor is a relatively small and straightforward instrument with no moving parts. The results show the successful production of monodispersed NPs on a large scale. NP size, which is the most crucial feature of NPs utilized in all of the biomedical applications, was readily adjusted by considering the process factors discussed in the paper, including the concentration and flow rate ratio of the feed streams. The concentration of feed streams had the most influence on the size of the final product. We also fabricated NPs that encapsulated a fluorescent dye, rhodamine 6G, on a large scale. The straightforward design and easy operation of the fiber reactor, together with the high level of control over NP size and PDI, make this system an excellent candidate for use in the pharmaceutical industry, for the scale up of nanomedicine formulations.

## Supplementary Materials

The following are available online at https://www.mdpi.com/1996-1944/13/14/3075/s1, Figure S1: Cross-section of one fiber and the streams that are flowing over the fiber and mathematical proof for calculation of mixing time.
