# Supplementary material for: High Throughput Preparation of Poly(Lactic-Co-Glycolic Acid) Nanoparticles Using Fiber Fluidic Reactor
Source: Materials (Basel). 2020 Jul 9;13(14):3075. doi: 10.3390/ma13143075 (PMC7411994; doi:10.3390/ma13143075)
Supplement: Supplementary file 1 [file materials-13-03075-s001.pdf]

materials

Supplementary Information

High Throughput Preparation of Poly(Lactic-Co-Glycolic Acid) Nanoparticles Using Fiber Fluidic Reactor

Niloofar Heshmati Aghda [1,†], Emilio J. Lara [2,†], Pulinkumar Patel [2] and Tania Betancourt [1,2,*]

[1] Materials Science, Engineering and Commercialization Program, Texas State University;, San Marcos, TX 78666, USA; n_h137@txstate.edu
[2] Department of Chemistry and Biochemistry, Texas State University, San Marcos, TX 78666, USA;ejl38@txstate.edu(E.J.L.); pulinpatel71@outlook.com(P.P.)
* Correspondence: tania.betancourt@txstate.edu; Tel.: +1-512-245-7703
† These authors contributed equally to this work.

Mathematical proof for calculation of mixing time (τ_{mix})

Assumptions:

- Mixing is diffusion-based only.
- Diffusion is unidirectional.
- Diffusion coefficient remains constant.
- Diffusion coefficient equals 1.4×10^{-5} cm²/s for acetone in water
- Distribution of the two phases along each single fiber are as illustrated in Figure S1
- Acetone stream width equals water stream width and they both equal diffusion width
- Fiber packing density is 8325 fibers/mm²
- Fiber diameter is 8 μm

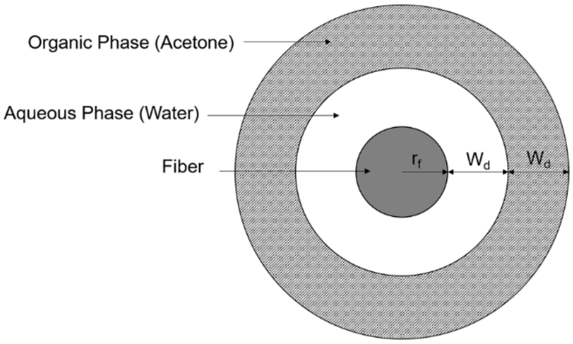

Figure S1. Cross-section of one fiber and the streams that are flowing over the fiber.

First, we calculate the one-directional distance associated with diffusion of the polymer solution into the aqueous phase, W_d. To do so, we first calculate the area occupied by fibers in an area of 1 mm²:

$$8325 \cdot \pi(4\ \mu m)^2 = 418{,}460\ \mu m^2$$

The void area in between fibers in a reactor area of 1 mm² is therefore:

$$(1000\ \mu m)^2 - 418{,}460\ \mu m^2 = 581{,}540\ \mu m^2$$

Therefore, the void area per fiber is:

$$581{,}540\ \mu m^2\ /\ 8325\ fibers = 69.85\ \mu m^2/fiber$$

From Figure S1, the area of one fiber and its void space can be described as:

$\pi(4\ \mu m)^2$ + $69.85\ \mu m^2 = \pi\ (2\ W_d\ /+\ r_f)^2$

where r_f is the fiber radius.

Solving for the unknow variable, we find that W_d = 1.09 μm.

In the second part of the derivation, we utilize Fick's second law of diffusion to find the time necessary to achieve full mixing of the polymer phase into the aqueous phase. According to the Fick's second law of diffusion:

$$\frac{\partial C}{\partial t} = D\frac{\partial^2 C}{\partial x^2} \qquad \text{Equation S1}$$

where C is volume fraction of acetone in the aqueous phase, which is a function of time (t) and distance (x), and D is the diffusion coefficient.

Boundary conditions are as follows:

At x = 0, C = 1 (for all t at the interface of two phases) B.C. S1

At t = 0, C = 0 (for all x in aqueous portion) B.C. S2

Therefore, the equation is solved as follows:

First, assume that

$$y = \frac{x}{2\sqrt{Dt}} \qquad \text{Equation S2}$$

Thus, we can rewrite equation 1 as

$$\frac{\partial C}{\partial y} = -\frac{1}{2y}\frac{\partial^2 C}{\partial y^2} \qquad \text{Equation S3}$$

And, the boundary conditions as:

At y = 0, C = 1 B.C. S3

At y = ∞, C = 0 B.C. S4

By defining

$$\frac{\partial C}{\partial y} = z \qquad \text{Equation S4}$$

Equation S3 can be written as

$$z = -\frac{1}{2y}\frac{\partial z}{\partial y} \qquad \text{Equation S5}$$

Then,

$$\int\frac{\partial z}{z} = -\int 2y\ \partial y \qquad \text{Equation S6}$$

Therefore,

$$ln\ z\ -\ ln\ cte_1 = -y^2 \qquad \text{Equation S7}$$

where cte_1 is an integration constant.

By rewriting the equation,

$$z = cte_1 . e^{-y^2} \qquad \text{Equation S8}$$

According to definition of z in equation S4

$$\frac{\partial C}{\partial y} = cte_1 . e^{-y^2} \qquad \text{Equation S9}$$

Thus,

$$\int \partial C = cte_1 . \int e^{-y^2} \partial y$$ Equation S10

For x in diffusion path:

$$\int_{C_{interface}}^{C(x,t)} \partial C = cte_1 . \int_{y_{interface}}^{y(x,t)} e^{-y^2} \partial y$$ Equation S11

Based on the first boundary condition (B.C. S1 and B.C. S3)

$$\int_{1}^{C(x,t)} \partial C = cte_1 . \int_{0}^{y(x,t)} e^{-y^2} \partial y$$ Equation S12

Thus,

$$C(x,t) - 1 = cte_1 . \int_{0}^{y(x,t)} e^{-y^2} \partial y$$ Equation S13

erf(y) is defined by

$$erf(y) = \frac{2}{\sqrt{\pi}} . \int_{0}^{y} e^{-y^2} \partial y$$ Equation S14

Then,

$$C(x,t) = 1 - cte_1 . \frac{\sqrt{\pi}}{2} erf(y)$$ Equation S15

Based on the second boundary condition (B.C. S2 and B.C. S4)

$$cte_1 = (0 - 1) . \frac{2}{\sqrt{\pi}} . erf(\infty) = \frac{-2}{\sqrt{\pi}}$$ Equation S16

Therefore,

$$C(x,t) = 1 - erf(y)$$ Equation S17

By substituting y from equation S2 in equation S17:

$$C(x,t) = 1 - erf\left(\frac{x}{2\sqrt{D.t}}\right)$$ Equation S18

To estimate the mixing time, the value of "C" is substituted with the final volume fraction of the two phases, "x" is substituted with the diffusion distance W_d, and the value of the diffusion coefficient is input to be able to solve for the time of mixing "t". For example, when C = 0.1, the mixing time will be 0.156 ms.
